# Rheological and Thermal Characterization of Kırkağaç Melon Seed Beverage Puddings Structured with Hydrocolloids

**DOI:** 10.3390/gels12090808

**Published:** 2026-09-03

**Authors:** Şelale Öncü Glaue

**Affiliations:** Food Processing Department, Efes Vocational School, Dokuz Eylül University, 35920 İzmir, Türkiye; selale.glaue@deu.edu.tr

**Keywords:** Kırkağaç melon seed beverage, by-product valorization, plant-based pudding, hydrocolloid, xanthan gum, galactomannan, κ-carrageenan, rheology, differential scanning calorimetry

## Abstract

Seeds from Kırkağaç melons (*Cucumis melo* L.), marketed locally as Kırkağaç Kavunu, are an underused co-product. This study evaluated whether a beverage obtained from these seeds could be structured into a plant-based pudding using xanthan gum, locust bean gum (LBG), κ-carrageenan or guar gum at 0.5 and 1.0% (*w*/*w*), without added starch. Starch-structured melon seed beverage and cow-milk puddings served as controls, and the formulations were characterized by rheology, differential scanning calorimetry (DSC) and attenuated total reflectance Fourier-transform infrared (ATR-FTIR) spectroscopy. Gum identity, concentration and their interaction significantly affected every rheological endpoint (interaction *p* ≤ 0.031), so equal mass fractions of different gums were not interchangeable structuring doses. Doubling the LBG concentration increased the loss modulus without increasing the storage modulus, reversing the balance between them, whereas doubling xanthan or guar strengthened their networks approximately threefold and ninefold, respectively. At 1 rad s^−1^ the storage modulus ranged from 3.68 ± 0.39 Pa for 0.5% guar to 475.40 ± 116.53 Pa for 1.0% κ-carrageenan, and four formulations showed a crossover between the storage (*G′*) and loss (*G″*) moduli within the measured frequency window. DSC resolved a reproducible transition at 68.2 ± 3.3 °C in the 0.5% κ-carrageenan pudding. Under the conditions tested, 0.5% κ-carrageenan provided the most balanced rheological response among the formulations examined, whereas the 1.0% gel was progressively expelled from the measuring gap under shear.

## 1. Introduction

Semi-solid dairy desserts are multicomponent soft solids in which proteins, lipids, starch, and non-starch polysaccharides jointly determine network formation, viscoelasticity, water immobilization, and oral texture. Replacing bovine milk with a plant-derived liquid is therefore not a simple substitution of the continuous phase: plant proteins, dispersed lipids, minerals and residual insoluble particles build different networks during heating and cooling. Studies on vegetable desserts, vegan rice puddings, rice-protein puddings, and pumpkin-seed-milk puddings show that botanical origin, liquid-phase composition, and stabilizer selection can alter storage modulus, viscosity, texture, and sensory acceptance [1,2,3,4,5,6].

Alongside this technological question sits a resource question. Fruit and vegetable processing generates large quantities of by-products, including peels, pomace and seeds, that are frequently discarded despite retaining nutritional and technological value [7,8]. Melon seeds contain substantial lipid, protein, carbohydrate and mineral fractions and have been investigated as value-added food ingredients [7,8,9]. Functional studies of *Cucumis melo* seed flour and defatted melon-seed fractions have reported water- and oil-absorption, emulsifying and foaming capacities, supporting their use as formulation ingredients rather than solely as oil sources [10,11].

*Kırkağaç kavunu* (*Kırkağaç melon*) is a geographically indicated melon associated with the Kırkağaç district of Manisa Province, Türkiye, and is valued for its distinctive aroma and unusually long shelf life. It was registered as a designation of origin by the Turkish Patent and Trademark Office on 12 January 2009 (registration no. 107, application no. C2006/031) and subsequently entered in the European Union register as a protected designation of origin (PDO), with publication on 25 July 2025 [12,13]. This status concerns the named fruit and its defined production area; it does not by itself establish superior nutritional or technological properties of the seed fraction, and the present work makes no such claim.

Melon seed beverages have previously been investigated as a means of converting an underused seed fraction into a liquid food ingredient. Karakaya et al. characterized the proximate composition of a beverage prepared from melon seeds [14], while Akubor and Ogbadu demonstrated that the processing method affects melon seed beverage quality and acceptability [15]. For Kırkağaç specifically, Bastıoğlu et al. prepared beverages from Kırkağaç and Çeşme seeds, reported pH and soluble solids values for the fresh liquids, and observed Bingham-type flow after spray-drying and reconstitution [9]. Their storage and sensory findings pertain to the spray-dried and reconstituted product and do not apply to the fresh puddings examined here. Together, these studies establish that melon seeds can yield a processable beverage, but they do not address the conversion of the seeds into a hydrocolloid-structured dessert gel.

Xanthan gum, LBG, κ-carrageenan and guar gum were selected because they structure aqueous systems through contrasting mechanisms. Xanthan is a highly shear-thinning anionic polysaccharide that forms weak, entangled networks; LBG and guar are galactomannans that differ in mannose-to-galactose ratio and hydration behavior and that thicken mainly by chain entanglement; and κ-carrageenan forms ion-mediated, thermoreversible double-helical junction zones that can yield firm and brittle gels [16,17,18,19,20,21,22,23,24,25]. Rheological studies further show that κ-carrageenan network response is sensitive to thermal and chemical history [26]. Binary-gel studies show that synergy is pairing-specific: κ-carrageenan and xanthan interact differently with individual galactomannans, while sucrose, ions, mixing ratio and total polymer concentration further modify the resulting network [27,28,29,30,31,32]. Pudding and dairy dessert studies consequently indicate that hydrocolloid identity, concentration, and matrix composition interact rather than acting as interchangeable mass fractions [1,2,5,6,16,27,33].

Previous studies have characterized melon seed beverages and plant-based puddings separately; however, the literature discussed above does not provide a matched, starch-free comparison of xanthan gum, locust bean gum, κ-carrageenan and guar gum in a fresh Kırkağaç melon seed beverage matrix. The present study therefore addresses both the feasibility of structuring this underused seed-derived beverage as a semi-solid food matrix and the gum type × concentration dependence of its rheological behavior. Accordingly, xanthan gum, LBG, κ-carrageenan and guar gum were compared at 0.5 and 1.0% (*w*/*w*) in puddings prepared from a Kırkağaç melon seed beverage, with starch-structured melon seed beverage and cow-milk formulations as reference systems. Rheological behavior was the primary structural outcome, while DSC and ATR-FTIR served as complementary thermal and spectroscopic characterization methods. The working hypothesis was that hydrocolloid identity and concentration would interact to produce distinct gel strengths, solid–liquid balances and shear-thinning profiles, such that equal mass fractions of different gums would not act as interchangeable structuring doses in this matrix.

## 2. Results and Discussion

### 2.1. Seed Processing Yield and Moisture Characteristics

Drying the washed seeds at 40 °C for 24 h reduced their mass from 137.24 to 77.91 g, a loss of 43.23% and a retained fraction of 56.77%. Gravimetric analysis of this pre-dried material gave a residual moisture content of 37.69 ± 3.41% and a dry-matter content of 62.31 ± 3.41% on a wet basis (Table 1). The 77.91 g therefore represents partially dried, not bone-dry, seed material; this distinction matters because the subsequent soaking and milling ratios were calculated from this pre-dried mass. The comparatively large coefficient of variation (9.1%) reflects the natural heterogeneity of the seed lot, in which individual seed moisture depends on seed size and coat integrity.

### 2.2. Melon Seed Beverage Composition

The proximate composition of the melon seed beverage was 86.33 ± 0.12% moisture, 13.67 ± 0.12% total dry matter, 1.30 ± 0.01% protein, 1.93 ± 0.05% fat, 0.25 ± 0.01% ash and 10.19 ± 0.07% carbohydrate on a wet basis (*n* = 3). Carbohydrate was calculated by difference. The pH was 7.025 ± 0.003 and the total soluble solids content was 3.41 ± 0.04 °Brix. The near-neutral pH and high-water content are relevant to hydrocolloid hydration and to the water-dominated ATR-FTIR spectra discussed below.

The pH and total soluble solids were close to the values of 7.04 and 3.5 °Brix reported for a Kırkağaç melon seed beverage by Bastıoğlu et al. [9]. The proximate profile was also comparable to that reported by Karakaya et al. [14]. Earlier work further showed that processing conditions affect the composition and acceptability of melon seed beverages [15]. These comparisons support the plausibility of the present measurements but do not establish product equivalence, as seed-to-water ratios and processing conditions differed across studies.

### 2.3. Macroscopic Gel Formation

K1 and K2 represent the starch-structured melon seed beverage and cow-milk controls, respectively. M, L, C and G refer to xanthan gum, locust bean gum, κ-carrageenan and guar gum, while the suffixes 1 and 2 indicate concentrations of 0.5 and 1.0% (w/w), respectively. After 12 h at 4 °C, all ten formulations formed visibly thickened, cohesive systems, and no macroscopic liquid separation was observed during visual examination. This observation was qualitative and was not treated as a quantitative syneresis measurement. Macroscopic appearance alone did not establish self-supporting gel behavior; the frequency sweeps subsequently identified four formulations as viscous-dominant below a characteristic frequency. The two starch controls, K1 and K2, were visually similar. Xanthan and LBG systems were also similar to the unaided eye despite their different mechanical spectra. Both guar formulations displayed a ropy, mucilaginous response during handling. The κ-carrageenan systems were the firmest: C1 remained loadable onto the rheometer, whereas C2 was extremely rigid and required a larger plate geometry (Section 4.7). This macroscopic firmness is consistent with the firm or brittle textures reported for κ-carrageenan-containing dessert and protein gels [16,18,19,27,34].

### 2.4. Frequency-Dependent Viscoelastic Behavior

**Figure 1:**
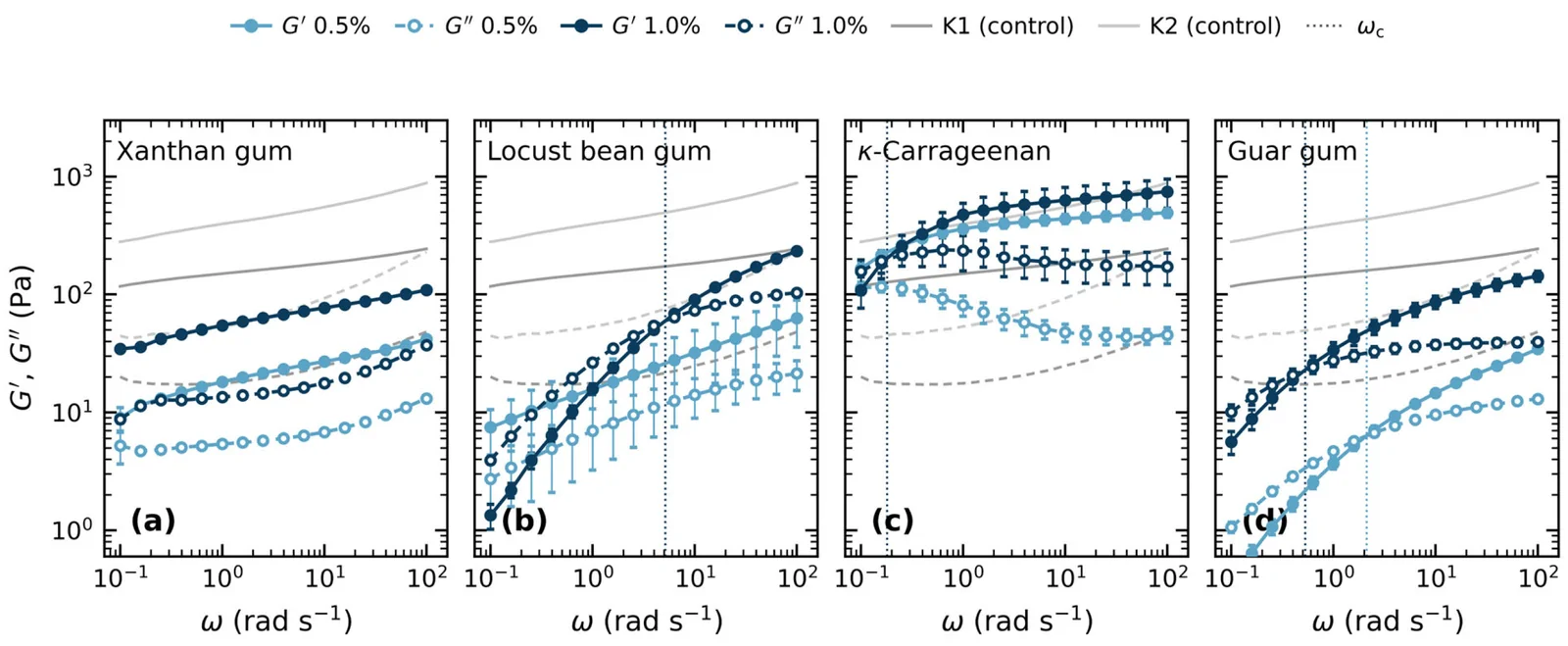
Mechanical spectra of the pudding formulations at 25 °C: (**a**) xanthan gum; (**b**) locust bean gum; (**c**) κ-carrageenan; and (**d**) guar gum. Storage modulus (*G′*, filled symbols, solid lines) and loss modulus (*G″*, open symbols, dashed lines) are plotted together on the same axes. Light and dark blue tones denote 0.5 and 1.0% gum, respectively. The dark- and light-grey reference curves correspond to the K1 and K2 controls, respectively; for the control curves, solid lines denote *G′* and dashed lines denote *G″*. All panels share identical axes. Symbols are means of three independent productions and error bars are standard deviations. Only the κ-carrageenan curves lie above the control references. Note the *G′*–*G″* crossovers in L2, G1, G2 and C2, which identify these systems as viscous-dominant below the frequencies given in Table 2.

The ten formulations occupied markedly different viscoelastic regimes (Figure 1, Table 2). At 1 rad s^−1^ the storage modulus spanned two orders of magnitude, from 3.68 ± 0.39 Pa for G1 to 475.40 ± 116.53 Pa for C2. Welch one-way analysis of variance, chosen because group variances were markedly unequal, detected highly significant formulation effects for log_10_ *G′* (*F* (9, 8.02) = 373.55), log_10_ *G″* (*F* (9, 8.04) = 221.57) and tan *δ* (*F* (9, 7.75) = 596.77; all *p* < 0.001), with identical conclusions from Kruskal–Wallis sensitivity analyses (*p* ≤ 0.001).

The starch controls behaved as expected for a gelatinized starch matrix: both were firmly solid-like, with loss tangents of 0.119 ± 0.011 (K1) and 0.135 ± 0.003 (K2) that did not differ significantly from one another (Holm-adjusted *p* = 0.108), while the cow-milk system was substantially stiffer than its plant-based counterpart (396.11 ± 85.14 versus 149.92 ± 40.51 Pa; adjusted *p* = 0.027). The difference may reflect the distinct solids composition and lower protein content of the seed beverage (1.30 ± 0.01%), together with the different protein–polysaccharide interactions present in the dairy matrix [19,35].

Among the gum-structured systems, tan *δ* provided a useful complementary measure of the balance between elastic and viscous responses and was interpreted together with the absolute moduli. C1 showed the lowest tan *δ*, whereas G1 and L2 exceeded unity at the reference frequency; the remaining formulations occupied intermediate elastic–viscous balances (Table 2). Four formulations exhibited a *G′*–*G″* crossover within the measured frequency window, demonstrating that classification at a single reference frequency did not fully describe their frequency-dependent behavior. G1 and L2 crossed over above the reference frequency. They consequently also showed loss tangents above unity at 1 rad s^−1^, whereas G2 and C2 crossed over below it and appeared elastic-dominant at the reference frequency. However, they did not maintain that dominance across the whole window. Neither guar formulation therefore maintained elastic dominance across the full measured frequency window at either concentration, while for LBG the crossover appeared only on doubling the concentration. This behavior is consistent with galactomannans that thicken through chain entanglement but do not independently form ordered junction zones [17,20,28,29,30,31,32].

C1 approached but did not reach a crossover, remaining elastic-dominant across the whole window (tan *δ* = 0.697 at 0.1 rad s^−1^). Because C2 was measured at 0.5% strain, about three times its strict linear limit of 0.16%, part of its elevated loss modulus may be non-linear in origin and its crossover frequency is reported as an upper-bound estimate rather than a relaxation time [36].

The frequency dependence of the storage modulus provides a descriptor of network persistence that does not rely on a single reference frequency. The exponent *n′* of the power-law fit of *G′* against angular frequency (Equation (1)) ordered the formulations along a continuum from weakly to strongly frequency-dependent (Table 2); a storage modulus that is almost independent of frequency indicates a network persisting over the measured range of timescales, whereas a strongly frequency-dependent modulus indicates a transient, entanglement-dominated response [37,38]. No universal numerical boundary separates these regimes, and the exponent is therefore used here as a relative ranking rather than as a categorical criterion. The exponent and the loss tangent ranked the ten formulations concordantly (Spearman ρ = 0.976, *p* < 0.001). Among the four formulations that crossed over, the crossover frequency ranked them in the same order as the exponent (ρ = 1.000), so three independent descriptors converged on a single ordering. K1 gave the least frequency-dependent modulus of the study (0.097 ± 0.016), followed by C1 (0.126 ± 0.013), K2, M2, M1 and C2; L1 and G2 were intermediate, and G1 and L2 the most strongly frequency-dependent. Gelatinized starch, therefore, produced the most persistent network in this matrix, even though κ-carrageenan produced the highest modulus. Two formulations were described less well by a single power law (*R*^2^ = 0.780 for C2 and 0.833 for C1, against *R*^2^ ≥ 0.940 for the others) because their storage moduli rose steeply below approximately 1 rad s^−1^ before flattening; restricted to 1–100 rad s^−1^ the same fits gave 0.065 ± 0.002 for C1 and 0.090 ± 0.016 for C2 with *R*^2^ ≥ 0.972, so both κ-carrageenan formulations became markedly less frequency-dependent at short timescales while relaxing at long ones. κ-Carrageenan produced the stiffest elastic-dominant networks among the gums: C1 reached *G′* = 359.79 ± 27.37 Pa and shared a homogeneous group with the cow-milk control (Games–Howell *p* > 0.05), whereas C2 gave the highest mean moduli of the study. The wide homogeneous group assigned to L1 reflects the dispersion of its storage modulus between independent productions (coefficient of variation 56%) rather than an intermediate network strength; the same replicates gave a frequency-dependence exponent with a coefficient of variation of 1.4%, suggesting that the variability mainly affected the absolute modulus rather than the frequency dependence of the response.

### 2.5. Steady-Shear Flow Behavior

**Figure 2:**
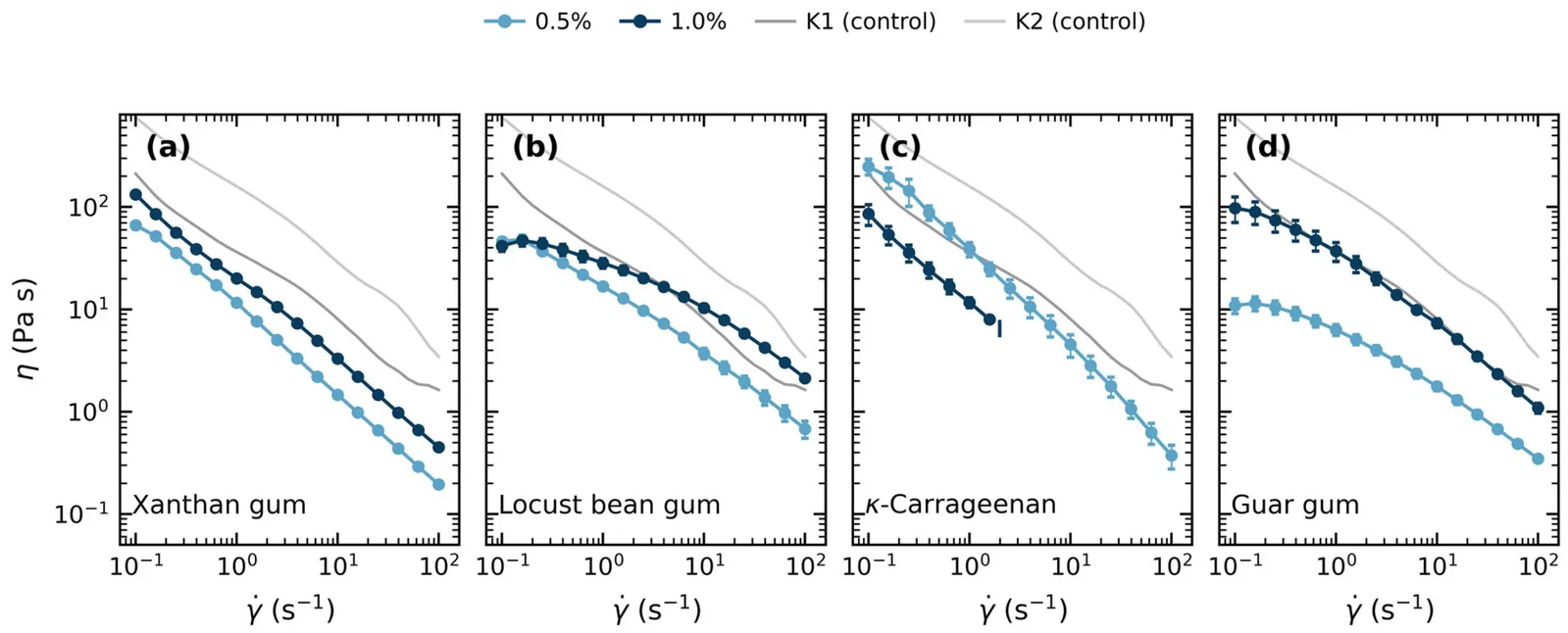
Steady-shear flow curves at 25 °C: (**a**) xanthan gum; (**b**) locust bean gum; (**c**) κ-carrageenan; and (**d**) guar gum. Light and dark blue tones denote 0.5 and 1.0% gum, respectively. The dark- and light-grey curves represent the K1 and K2 controls, respectively. Symbols are means of three independent productions and error bars are standard deviations. The C2 curve is shown only up to 1.59 s^−1^, the highest shear rate within the common interval over which the normal force remained within 20% of its plateau value in all three replicates; above this range, the sample was progressively expelled from the measuring gap.

Flow curves were recorded from 0.1 to 100 s^−1^ (Figure 2, Table 3). Every formulation was markedly shear-thinning over its technically valid branch. Apparent viscosity at 1 s^−1^ differed significantly among formulations (Welch F(9, 7.46) = 112.72, *p* < 0.001), with K2 showing the highest value and G1 the lowest; the remaining formulations were intermediate (Table 3).

Flow curves were described by the Ostwald–de Waele model, in which no yield stress was resolved, and by the Herschel–Bulkley model, in which one was. Model selection followed the criterion that the fitted yield stress exceeded its own standard deviation across replicates. Eight formulations were adequately described by the power law (*R*^2^ ≥ 0.945), with flow behavior indices ranging from 0.133 to 0.550, confirming pronounced pseudoplasticity throughout; the two κ-carrageenan formulations required separate treatment. For C1 the shear stress was almost independent of shear rate, rising only 1.6- to 1.9-fold between 0.1 and 100 s^−1^. A single power law described these curves poorly (*R*^2^ = 0.501). Constrained Herschel–Bulkley fitting drove the yield stress to zero across all three replicates because *τ*_0_ and K become mathematically inseparable as the flow index approaches zero. No flow model, therefore, provided a meaningful parameterization of C1, and its flow parameters are reported as not determined; only the directly measured apparent viscosity at 1 s^−1^ is given. For C2, the sample was progressively expelled from the measuring gap under shear, as indicated by the normal force, which first fell below 80% of its plateau value at 4.0, 2.0, and 2.5 s^−1^ across the three replicates. Flow curves were therefore evaluated over the common interval within which the normal force remained within 20% of its plateau in every replicate, that is up to and including 1.59 s^−1^. Herschel–Bulkley fitting of this branch resolved a yield stress of 6.76 ± 1.73 Pa with a consistency coefficient of 4.76 ± 0.77 Pa·s*^n^* and a flow behaviour index of 0.529 ± 0.128 (*R*^2^ = 0.980); including the next shear rate, at which one replicate had already begun to lose sample, lowered the estimate to 5.97 ± 1.27 Pa, and extending the interval further doubled its dispersion. The two κ-carrageenan systems therefore lost structural integrity under shear by different routes: at 0.5% through a near-constant-stress response that no model captures, and at 1.0% through expulsion from the gap. Because cross-hatched plates were used, simple wall slip at the plate surface is a less likely primary explanation for these responses, which are more consistent with failure within the sample itself: a near-continuous yielding of the 0.5% network and macroscopic expulsion of the 1.0% gel. Neither behavior is compatible with a stable equilibrium flow curve, and neither formulation should be characterized by flow parameters alone [39].

Across the data set, the flow behavior index was inversely related to every viscoelastic modulus within the power-law formulations, for which the index is defined on a common basis (Section 2.9), indicating that the formulations with the stiffest networks also showed the steepest decrease in apparent viscosity with increasing shear rate. This relation is relevant to processing behavior, although spoonability and pumpability would require dedicated instrumental or sensory measurements.

### 2.6. Interaction Between Gum Type and Concentration

Because the eight gum formulations form a balanced 4 × 2 design, they were additionally analyzed by two-factor analysis of variance (Table 4). Gum type, concentration and critically their interaction were significant for every outcome (interaction *p* ≤ 0.031), with very large effect sizes for the interaction term in log_10_ *G′* (partial η^2^ = 0.814), tan *δ* (0.979) and log_10_ viscosity (0.966). Equal gum mass fractions therefore cannot be treated as equivalent structuring doses: the concentration response was strongly gum-dependent.

**Figure 3:**
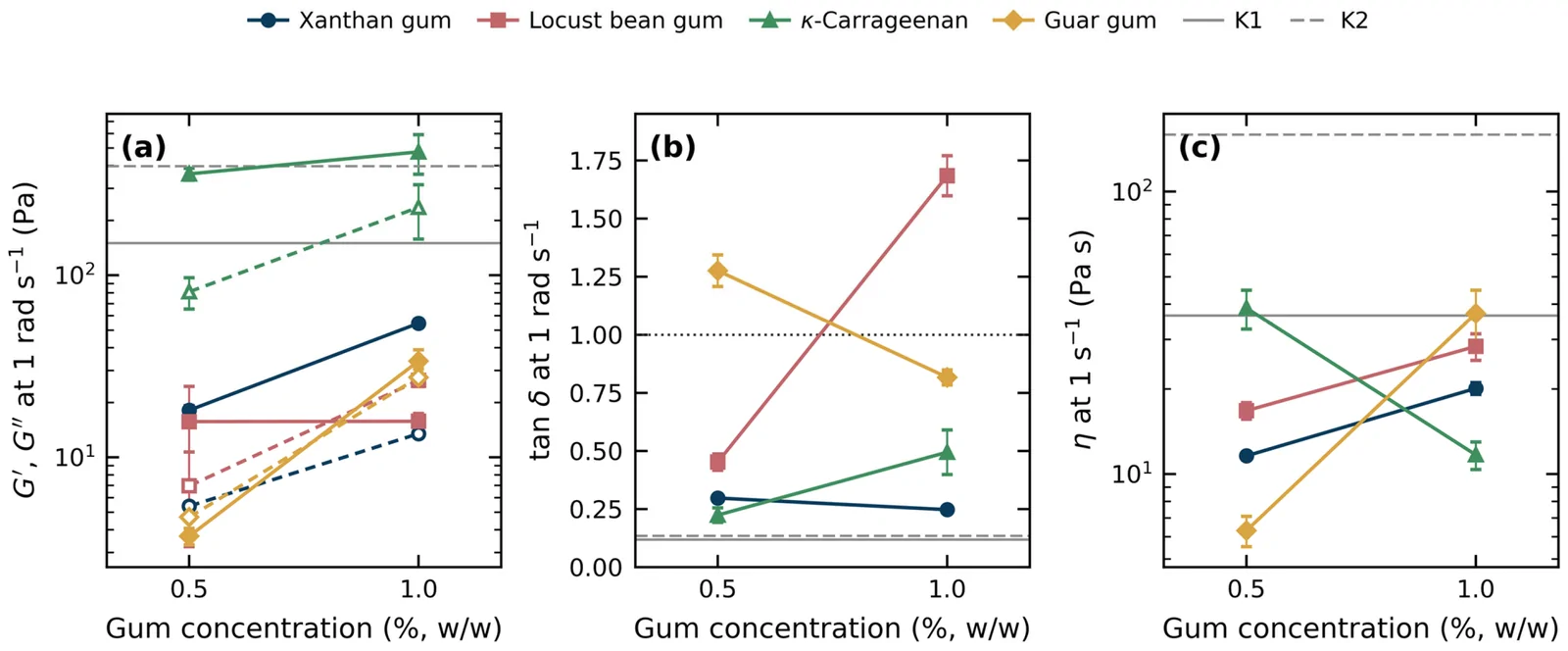
Effect of hydrocolloid concentration on the principal rheological responses, one line per gum: (**a**) storage and loss moduli at 1 rad s^−1^ (*G′*, filled symbols and solid lines; *G″*, open symbols and dashed lines), (**b**) loss tangent at 1 rad s^−1^, and (**c**) apparent viscosity at 1 s^−1^. Symbols are means and whiskers are standard deviations of three independent productions. Non-parallel lines indicate the gum × concentration interaction reported in Table 4. The horizontal lines mark the two starch-structured controls; in panel (**a**), these reference lines correspond to the control *G′* values. The dotted line in panel (**b**) marks tan *δ* = 1, indicating the transition between elastic-dominant and viscous-dominant behavior at the reference frequency.

Figure 3 displays this interaction directly: the lines describing the four gums are clearly non-parallel, and in panel (b) the LBG and guar lines cross the tan *δ* = 1 boundary in opposite directions. The planned within-gum contrasts make this concrete. Raising xanthan from 0.5 to 1.0% increased *G′* approximately threefold and guar approximately ninefold (both Holm-adjusted *p* ≤ 0.001). In contrast, LBG showed no change in *G′* (adjusted *p* = 0.771) yet a significant increase in *G″* (adjusted *p* = 0.044) and in tan *δ* (adjusted *p* = 0.002). LBG thus illustrates most clearly that concentration can shift the solid–liquid balance of a system without strengthening it, an outcome consistent with the non-additive interactions and phase-separation behavior reported for galactomannan-containing polysaccharide systems [28,29,30,31,32,33]. For κ-carrageenan the difference in *G′* between 0.5 and 1.0% was not significant (adjusted *p* = 0.373). However, *G″* increased (adjusted *p* = 0.028), so the extra gum contributed mainly to viscous dissipation rather than to elastic structure. Apparent viscosity at 1 s^−1^ changed significantly with concentration for all four gums (adjusted *p* ≤ 0.006).

### 2.7. Thermal Behavior

Because every pudding had already been heated to 85 °C during manufacture, a residual starch-gelatinization endotherm was not expected on the first DSC heating scan, and none was resolved. In the starch controls and in all xanthan, LBG and guar formulations, the first heating curve between 5 and 100 °C was featureless within the detection criteria, with departures from the linear baseline below three times the noise standard deviation. The absence of a resolvable transition in K1 is consistent with extensive starch gelatinization during processing; however, it does not, by itself, establish complete gelatinization or exclude overlapping low-enthalpy transitions in the melon seed beverage matrix.

The absence of a resolved transition in the galactomannan and xanthan formulations is not, in itself, unexpected. Locust bean gum and guar gum are essentially disordered coil polymers that thicken through chain entanglement rather than through cooperative junction zone formation, so no order–disorder transition is anticipated for them over the temperature range examined [17,20,28,29,30,31,32]. Xanthan does undergo a thermally induced conformational order–disorder transition, but its midpoint temperature depends strongly on ionic strength and counterion activity [40], so the present result indicates only that the associated enthalpy fell below the resolution criterion applied in this matrix, not that the transition does not occur. Detection sensitivity is a further consideration: the total hydrocolloid mass in each pan was only 65–130 µg in a matrix containing more than 80% water, which may have limited the detectability of low-enthalpy transitions.

**Figure 4:**
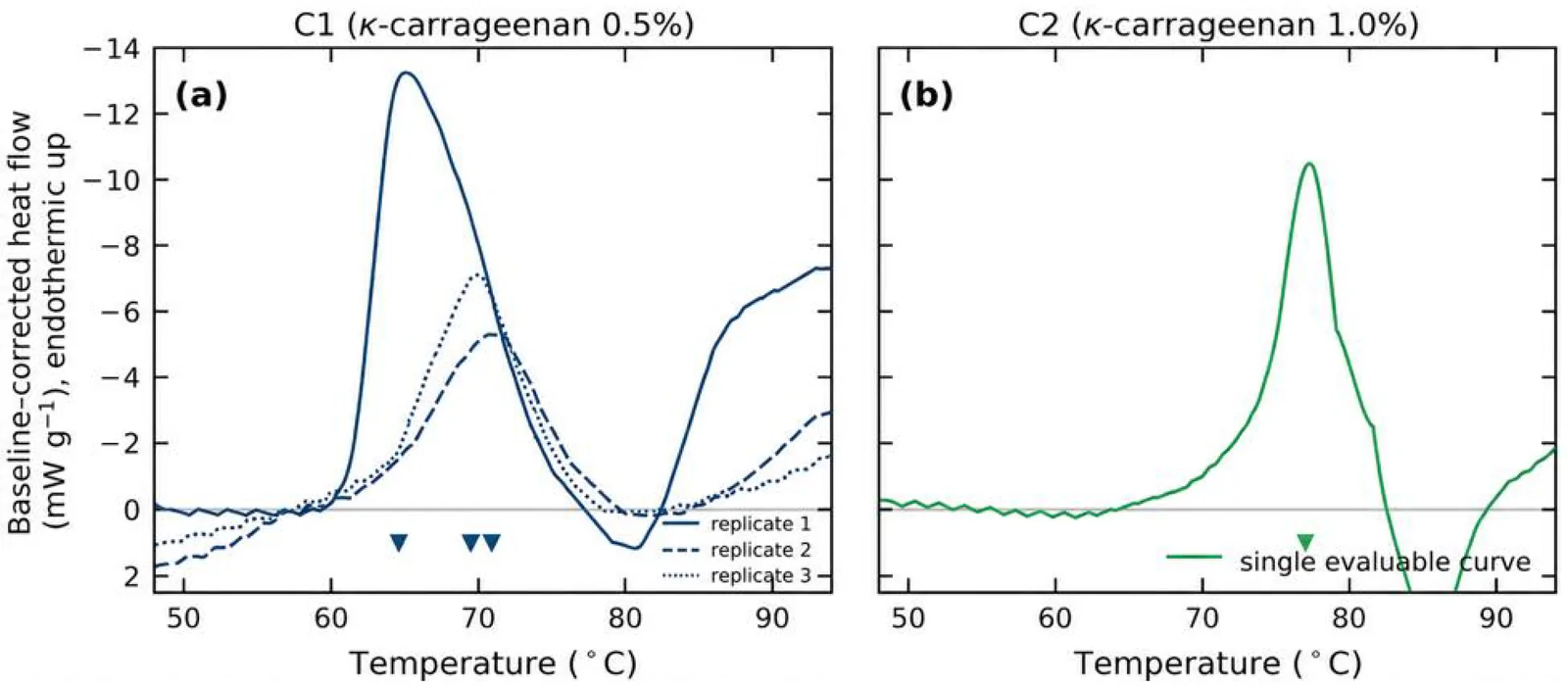
DSC thermograms of the κ-carrageenan formulations (10 °C min^−1^, endothermic upward, locally baseline-corrected and smoothed for display): (**a**) the three evaluable first-heating curves of C1; (**b**) the single evaluable first-heating curve of C2. Triangles indicate the peak temperatures of the individual thermograms.

The κ-carrageenan formulations were the only ones showing a resolvable, formulation-associated transition (Figure 4, Table 5). C1 displayed a broad first-heating endotherm with an onset of 65.1 ± 2.6 °C and a peak of 68.2 ± 3.3 °C at a signal-to-noise ratio of 23, against a ratio of 1.5 obtained by applying the same procedure to a control window between 25 and 45 °C in which no transition is expected. The single evaluable C2 thermogram showed a transition about 9 °C higher, with an onset of 75.4 °C and a peak of 77.2 °C; because this rests on one curve it is reported as an observation rather than as a demonstrated concentration-dependent shift, and the peak position was insensitive to the choice of integration window, appearing between 76.9 and 77.2 °C for half-widths from 8 to 12 °C.

The single evaluable C2 curve gave a lower apparent enthalpy per gram of κ-carrageenan (29 J g^−1^) than the C1 mean (84 ± 38 J g^−1^), but no concentration trend can be inferred from one curve. Both values fall within the range reported for κ-carrageenan helix–coil transitions, but the decrease should be interpreted with caution: only one C2 thermogram was evaluable, its integration was sensitive to the window width, and the total polymer mass in a pan was only 65–130 µg. Concentration, ionic environment, cosolutes and associated polysaccharides can all shift κ-carrageenan sol–gel transitions and junction-zone stability [18,21,22,23,24,25]. DSC alone cannot establish the molecular origin of the shift. Second-heating scans were recorded after controlled cooling to 5 °C but are not reported: under the stated evaluation protocol the peak temperatures were not reproducible between replicates, and no statement about thermoreversibility is therefore made.

### 2.8. ATR-FTIR Spectroscopic Characterization

**Figure 5:**
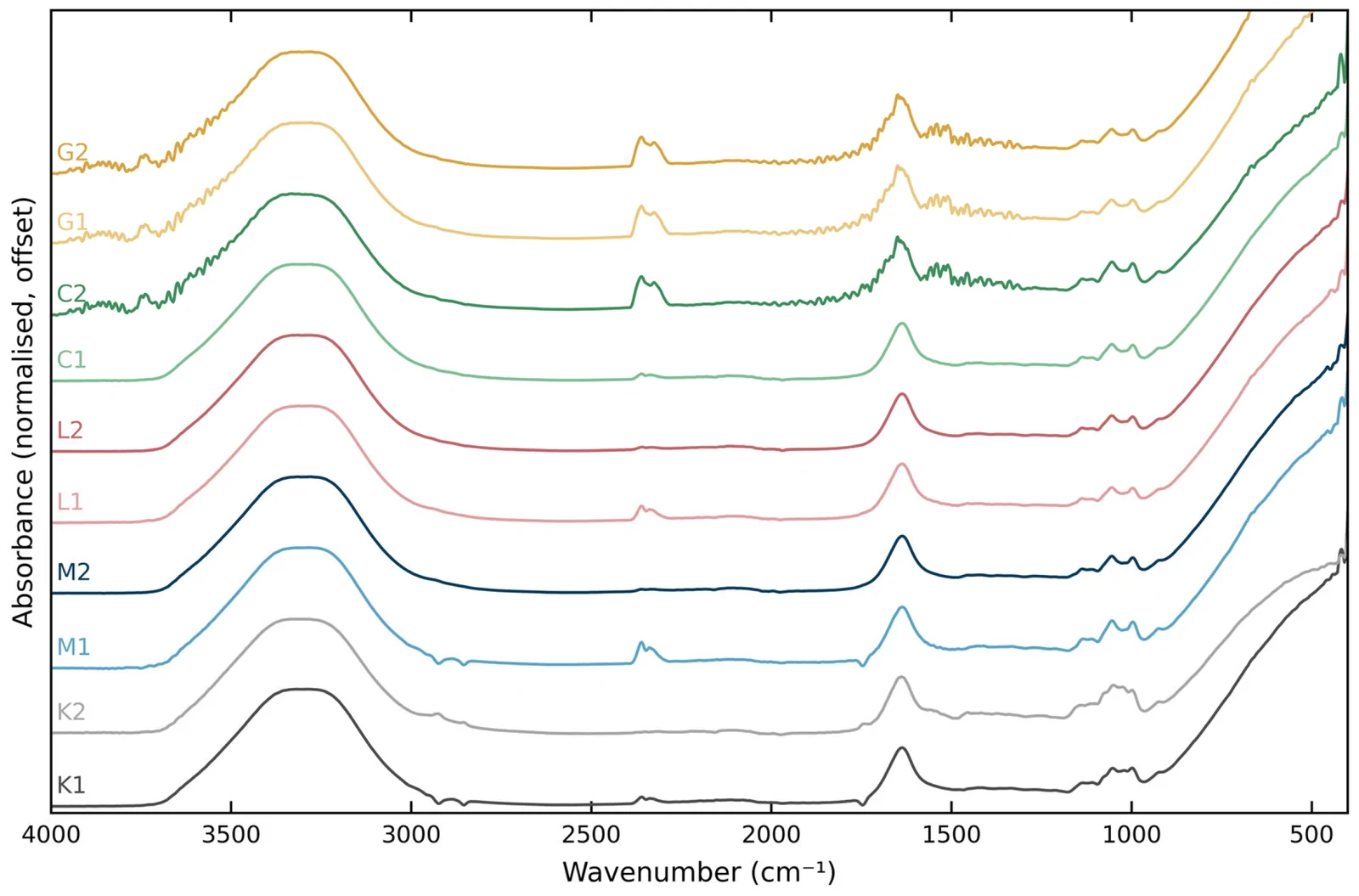
ATR-FTIR spectra of the ten formulations (group-mean spectra from three independent pudding productions, normalized on the O–H stretching envelope and vertically offset). Spectra are shown after Savitzky–Golay smoothing for display only (11 points, approximately 21 cm^−1^, third order); all quantitative comparisons were performed using the unsmoothed spectra. All spectra are dominated by the O–H stretching and H–O–H bending bands of water, and no distinct new bands or reproducible band shifts were resolved after hydrocolloid addition.

ATR-FTIR spectra were reproducible within each formulation and qualitatively similar across formulations (Figure 5). Every spectrum was dominated by the broad O–H stretching envelope centered near 3280–3310 cm^−1^ and the H–O–H bending band at 1635–1647 cm^−1^, as expected for systems containing more than 80% water. In three formulations (C2, G1, and G2), the spectra also carried uncompensated atmospheric water-vapor and carbon dioxide bands, identified by residual absorbance at 2320–2380 cm^−1^ that was three- to twentyfold higher than in the remaining spectra and by an apparent 11.6 cm^−1^ displacement of the water bending maximum. Because these features are instrumental rather than compositional, quantitative comparisons were restricted to the 800–1300 cm^−1^ polysaccharide region, in which all ten spectra were free of atmospheric interference.

Within that region, spectral differences were quantified as the mean absolute difference between vector-normalized group means and compared with the corresponding dispersion between replicates of the same formulation, which averaged 0.0021 in the same units. Doubling the gum concentration changed the normalized spectrum by 0.2 times this replicate dispersion for guar, 1.0 times for LBG, 3.1 times for xanthan and 4.4 times for κ-carrageenan. The only differences that clearly exceeded replicate dispersion were those between the cow-milk control and the plant-based formulations (10 to 12 times), consistent with the different protein and fat composition of the two liquid bases rather than with hydrocolloid identity. No distinct new absorption bands or reproducible band shifts attributable to hydrocolloid addition were resolved. This finding should not be treated as evidence that molecular interactions were absent. Strong water absorption, overlap between the H–O–H bending and amide I regions, and sampling-dependent spectral intensity all limit the sensitivity of ATR-FTIR in high-moisture systems of this type [41,42,43,44]. Hydration can also alter polysaccharide spectra and their multivariate discrimination [45,46], and quantitative applications of infrared spectroscopy to liquid foods generally require controlled sampling, spectral subtraction or purpose-built calibration [47].

The characteristic κ-carrageenan marker bands at approximately 930 cm^−1^ (3,6-anhydro-D-galactose) and 845 cm^−1^ (D-galactose-4-sulfate) [48,49] were not resolved above the replicate dispersion. Second-derivative inspection of the sulfate-ester region near 1240 cm^−1^ [50] likewise did not provide carrageenan-specific discrimination, because comparable features occurred in several other formulations. Thus, ATR-FTIR did not independently verify the carrageenan network inferred from rheology and DSC and is interpreted here only as supportive comparative evidence.

### 2.9. Integrated Structure–Property Relationships and Formulation Selection

**Figure 6:**
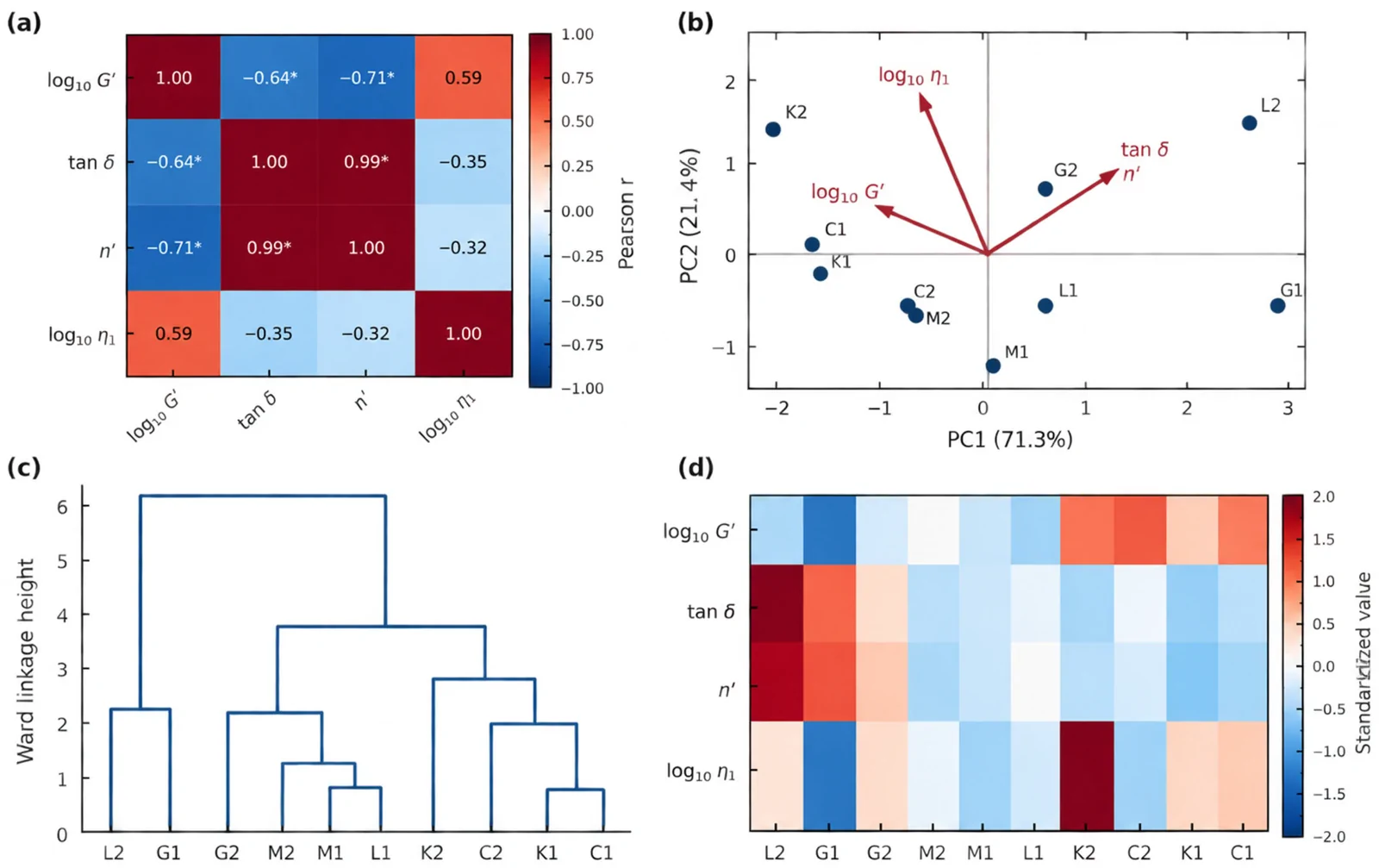
Multivariate analysis of the rheological data: (**a**) Pearson correlation matrix of the four descriptors, with asterisks marking significant coefficients (* *p* < 0.05); (**b**) principal component analysis biplot of the ten formulations, with red arrows showing variable loadings; (**c**) hierarchical clustering dendrogram (Ward’s method, Euclidean distance); and (**d**) heatmap of the standardized descriptors ordered by the dendrogram. The ordination and clustering are presented as an exploratory summary of the covariance structure of the formulation means rather than as inferential results.

Rheology gave the most reproducible discrimination among the formulations. Restricting the multivariate analysis to four non-redundant descriptors (log_10_ *G′*, tan *δ*, *n′* and log_10_ η at 1 s^−1^) avoided the definitional dependencies that would otherwise dominate the covariance structure. The strongest association was between tan *δ* and *n′* (r = 0.987, *p* < 0.001), while log_10_ *G′* was inversely related to both variables (Figure 6a).

The first two principal components explained 92.7% of the total variance (PC1, 71.3%; PC2, 21.4%) (Figure 6b). PC1 primarily separated formulations according to network persistence. L2 and G1 occupied the positive extreme, consistent with their high tan *δ* and *n′* values, whereas K1 and C1 were positioned on the negative side and were characterized by low frequency dependence and elastic-dominant responses. K2 was also located at the negative side of PC1 but separated strongly along PC2, reflecting its high apparent viscosity. G2 occupied an intermediate position, while M1, M2, L1 and C2 showed intermediate combinations of stiffness, damping and consistency.

Hierarchical clustering provided a complementary grouping of the formulations (Figure 6c). L2 and G1 formed a distinct branch separated from the remaining systems, consistent with their high damping and frequency dependence. M1, M2, L1 and G2 occupied an intermediate cluster, whereas K1 and C1 were closely associated within the more persistent-network group; K2 and C2 joined this broader branch at greater linkage distances. The standardized heatmap (Figure 6d) confirms these relationships: L2 and G1 show high standardized tan *δ* and *n′* values, K1 and C1 combine relatively high *G′* with low damping and frequency dependence, K2 is distinguished particularly by high viscosity, and the remaining gum formulations show intermediate multivariate profiles.

Translating these results into a formulation recommendation requires distinguishing the stiffest gel from the most workable one. C2 gave the largest mean storage modulus of the study, a storage modulus that was only weakly frequency-dependent above 1 rad s^−1^, and a resolvable yield stress over a restricted range of shear rates. Its drawbacks were nevertheless substantial: it was extremely rigid and difficult to load, required a larger measuring geometry, became viscous-dominant below 0.18 rad s^−1^ and was expelled from the measuring gap above approximately 1.6 s^−1^.

C1 delivered a storage modulus of 359.79 ± 27.37 Pa and shared a homogeneous statistical group with the cow-milk control (Games–Howell *p* > 0.05); this absence of a detected difference does not establish equivalence. It remained elastic-dominant across the entire measured frequency window, which C2 did not, and remained loadable and measurable throughout. C1 is therefore identified as the most balanced rheological candidate among the formulations tested, whereas C2 represents the stiffest but less workable system. C1 yielded no interpretable flow-model parameters, whereas C2 yielded an apparent yield stress only over the restricted pre-expulsion branch. M2 represents a rational third option for a less rigid system: it formed a moderate, elastic-dominant network (54.49 ± 2.70 Pa; tan *δ* = 0.247 ± 0.001) without the ropy handling character of the guar systems or the rigidity of C2. At larger processing scales, uniform hydrocolloid dispersion, heat transfer and shear history during pumping and filling would require validation. The shear instability observed for C2 suggests that high κ-carrageenan levels may present handling challenges during scale-up, whereas C1 provides a more tractable starting point for pilot-scale evaluation.

Several limitations bound these conclusions. Melons marketed locally as Kırkağaç Kavunu were used; however, harvest year and lot identifiers could not be documented because the fruits were purchased as unpackaged produce, and PDO certification of the specific experimental lot was not independently verified. The extraction ratios were calculated from partially dried rather than dry-matter seed mass, which limits reproducibility across seed lots. The three replicates of each formulation represent independent productions from separately prepared beverage batches, but a single raw-material lot was used, so they constitute process rather than biological replication. Accordingly, both the absolute rheological values and the relative formulation ranking should be confirmed across independent seed lots, harvest seasons and production locations before broader generalization. No instrumental texture profile analysis, syneresis measurement, storage study or sensory evaluation was performed, so the recommendation of C1 is technological rather than consumer based. Only one of three C2 thermograms met the predefined quality criteria; the other two showed a change in baseline heat flow between heating scans consistent with water loss. The C2 thermal transition therefore rests on a single curve. C2 also required a larger measuring geometry because the rigid gel could not be loaded reproducibly at the smaller gap, so its rheological data are not strictly geometry-matched to the other formulations; the measurement conditions are reported in full so that the comparison can be judged. Cross-hatched plates were used to reduce wall slip, which makes plate-surface slip less likely as the primary explanation for the loss of structural integrity in the κ-carrageenan formulations, although it cannot be excluded. Three ATR-FTIR spectra (C2, G1 and G2) carried uncompensated atmospheric bands, so the spectroscopic comparison was restricted to the 800–1300 cm^−1^ region, and the water-dominated spectra limit the corresponding inferences. These limitations define the experiments needed before product optimization or any consumer claim.

## 3. Conclusions

A beverage prepared from the seeds of melons marketed as Kırkağaç Kavunu (Kırkağaç melon) can serve as the continuous phase of a semi-solid pudding matrix. Still, hydrocolloid identity and concentration produced fundamentally different rheological states rather than graded versions of the same structure. The starch-structured plant control was elastic-dominant (tan *δ* = 0.119 ± 0.011) but had a storage modulus approximately 2.6-fold lower than that of its cow-milk counterpart.

Among the four gums evaluated without added starch, C1 reached a storage modulus of 359.79 ± 27.37 Pa and did not differ significantly from the cow-milk reference in the Games–Howell comparison; this absence of a detected difference does not establish material equivalence. Unlike C2, C1 remained elastic-dominant across the measured frequency window and could be loaded and characterized under the standard geometry. It was also associated with a reproducible DSC transition at 68.2 ± 3.3 °C; the single evaluable thermogram of the 1.0% formulation showed a transition at a higher temperature, 77.2 °C, but a concentration-dependent shift cannot be established from one curve. Doubling the concentration produced the stiffest network of the study and a resolvable yield stress over a restricted range of shear rates, but also a structure that became viscous-dominant below 0.18 rad s^−1^ and was expelled from the measuring gap under shear. The higher dose therefore yielded the stiffer but less workable formulation, whereas the lower dose provided the more balanced rheological response.

Gum type, concentration and their interaction were significant for every rheological endpoint, with very large interaction effect sizes. Increasing xanthan or guar strengthened their networks roughly threefold and ninefold, respectively, whereas increasing LBG raised the loss modulus without raising *G′* and converted the system to viscous-dominant behavior. Equal mass fractions of different gums are therefore not interchangeable when structuring doses in this matrix.

ATR-FTIR did not resolve distinct new absorption bands or reproducible band shifts after hydrocolloid addition. Because water dominated the spectra, this result does not establish the presence or absence of specific molecular interactions and is used only as supporting comparative evidence. Future work should address instrumental texture, quantitative syneresis and refrigerated storage stability. Binary hydrocolloid systems, particularly κ-carrageenan/galactomannan combinations, represent a logical next step for future formulation development. Within these bounds, 0.5% κ-carrageenan is the most promising rheological starting formulation among those tested; however, instrumental texture, syneresis, storage and sensory validation are required before product optimization.

## 4. Materials and Methods

### 4.1. Materials and Raw Material Provenance

Mature melons marketed locally as Kırkağaç Kavunu (Kırkağaç melon) were purchased as fresh, unpackaged produce from a local market in Manisa, Türkiye. Kırkağaç Kavunu is a registered designation of origin in Türkiye and a protected designation of origin (PDO) in the European Union [12,13]. However, because the fruits used in this study were sold unpackaged, individual fruit labels, harvest-year information and lot numbers were not available, and PDO certification of the specific experimental lot was not independently verified. Food-grade sucrose and corn starch were purchased locally. Food-grade xanthan gum (E415), guar gum (E412), locust bean gum (E410) and fully refined κ-carrageenan (E407) were supplied by Benosen Gıda San. Kimya ve Dış Tic. Ltd. Şti. (Istanbul, Türkiye) and used as received. The nominal viscosities of the guar gum and locust bean gum were 5500 and 3200 cP, respectively. The κ-carrageenan was buffer-free and had a specified gel strength of 1200–1800 g cm^−2^ in 0.2% KCl at 20 °C. Full-fat UHT cow milk from a local market served as the dairy reference. Distilled water was used throughout beverage and pudding preparation.

### 4.2. Preparation and Moisture Determination of Melon Seeds

Seeds were separated manually from the fruit flesh of freshly purchased melons, washed with tap water to remove adhering pulp and dried in a forced-air oven (Memmert GmbH + Co. KG, Schwabach, Germany) at 40 °C for 24 h. The pre-dried seeds were not stored. Subsamples were taken immediately for residual-moisture determination, and the remaining material was used directly for subsequent beverage preparation. Seed mass before and after this pre-drying step was 137.24 and 77.91 g, respectively. Residual moisture was then determined gravimetrically in triplicate on the pre-dried material by drying to constant mass. Dry matter (%) was calculated as 100 × dry sample mass/initial sample mass and wet-basis moisture (%) as 100 − dry matter.

### 4.3. Preparation of the Melon Seed Beverage

The beverage-preparation procedure was based on previously reported melon-seed beverage methods [9,14,15], with modifications in seed pre-drying, soaking conditions, seed-to-water ratio, homogenization and clarification. Three independent beverage batches were prepared. Pre-dried seeds were immersed in distilled water at a ratio of 1 g seed to 5 mL water and held at room temperature for 8 h, after which the soaking water was discarded. The swollen seeds were combined with fresh distilled water at a ratio of 1 g to 8 mL, with the second ratio calculated from the original pre-dried seed mass rather than the swollen mass. The mixture was homogenized in a Waring blender (Waring Commercial, Stamford, CT, USA) at maximum speed for 3 min, filtered through cheesecloth, and centrifuged at 5000 rpm for 10 min at room temperature; the rotor radius and the corresponding relative centrifugal force were not recorded. The clear supernatant was collected as the plant-based beverage and used immediately.

### 4.4. Characterization of the Melon Seed Beverage

Moisture, crude protein, crude fat and ash were determined in triplicate according to standard AOAC procedures using oven drying, Kjeldahl analysis (N × 6.25), Soxhlet extraction and dry ashing, respectively [51]. Total dry matter was calculated as 100 − moisture, and carbohydrate was calculated by difference as carbohydrate (%) = 100 − [moisture (%) + protein (%) + fat (%) + ash (%)]. The pH was measured at room temperature using a calibrated pH meter (Thermo Fisher Scientific, Waltham, MA, USA). Total soluble solids were determined using a refractometer (ATAGO Co., Ltd., Tokyo, Japan) and expressed as °Brix. Measurements were performed separately for the three independent beverage batches.

### 4.5. Experimental Design and Pudding Formulations

Ten formulations of 100 g each were prepared (Table 6). The two controls were structured with corn starch: K1 contained 84 g melon seed beverage, 8 g sucrose, 3 g water and 5 g corn starch, and K2 was identical except that the beverage was replaced by 84 g full-fat UHT cow milk. The eight gum formulations contained no added starch: the 0.5% series comprised 84 g beverage, 8 g sucrose, 7.5 g water, and 0.5 g gum, and the 1.0% series comprised 84 g beverage, 8 g sucrose, 7 g water, and 1 g gum. Xanthan gum (M1/M2), locust bean gum (L1/L2), κ-carrageenan (C1/C2) and guar gum (G1/G2) were evaluated. The common concentration levels of 0.5 and 1.0% (w/w) were selected deliberately to enable a matched mass-fraction comparison across hydrocolloids rather than to optimize each gum individually. This design allowed the gum type × concentration interaction to be tested directly and was not intended to imply equivalent functional potency of the four hydrocolloids. The design thus contains a starch-structured reference pair and a balanced 4-gum × 2-concentration factorial of gum-structured systems; the two structuring principles are compared descriptively rather than as a single factorial. Binary hydrocolloid mixtures were intentionally excluded from the factorial design because the objective was first to establish gum-specific concentration responses under matched single-hydrocolloid conditions. Pairing-specific synergism or phase separation would introduce an additional compositional variable and make attribution of individual gum and concentration effects less direct [18,20,27,28,29,30,31,32,52]. Each formulation was produced from three independently prepared beverage batches, giving three independent pudding batches per formulation.

**Table 6:** Formulation codes and composition of the control and hydrocolloid-structured pudding formulations (g per 100 g).

Code	Liquid Base (84 g)	Sucrose	Water	Structuring Agent
K1	Melon seed beverage	8	3.0	5.0 g corn starch
K2	Full-fat UHT cow milk	8	3.0	5.0 g corn starch
M1	Melon seed beverage	8	7.5	0.5 g xanthan gum
M2	Melon seed beverage	8	7.0	1.0 g xanthan gum
L1	Melon seed beverage	8	7.5	0.5 g locust bean gum
L2	Melon seed beverage	8	7.0	1.0 g locust bean gum
C1	Melon seed beverage	8	7.5	0.5 g κ-carrageenan
C2	Melon seed beverage	8	7.0	1.0 g κ-carrageenan
G1	Melon seed beverage	8	7.5	0.5 g guar gum
G2	Melon seed beverage	8	7.0	1.0 g guar gum

No starch was added to the gum-structured formulations; each formulation totals 100 g.

### 4.6. Pudding Preparation

The pudding-processing procedure was developed with reference to previously reported plant-based pudding formulations [2,6], with modifications to the formulation, hydrocolloid dispersion sequence and heating conditions. For the gum formulations, the gum and sucrose were dry-blended, sprinkled slowly into a portion of the beverage, briefly allowed to wet, and mixed to disperse and hydrate the gum before the remaining beverage and water were added. In the starch controls, starch and sucrose were added directly without the dry pre-blend step. Each batch was processed in a Thermomix TM6 (Vorwerk, Wuppertal, Germany) using a three-stage program: homogenization for 2 min at speed 3, heating at 85 °C for 12 min at speed 3 and holding at 85 °C for 3 min at speed 2. Samples were held at room temperature for 15 min and then stored at 4 °C for 12 h before analysis.

### 4.7. Rheological Measurements

Measurements were performed on a Discovery HR-20 rheometer (TA Instruments, New Castle, DE, USA) with Peltier temperature control and a solvent trap, at 25 °C. Nine formulations (K1, K2, M1, M2, L1, L2, G1, G2 and C1) were measured with a 40 mm parallel plate at a 500 µm gap; C2 was measured with a 50 mm parallel plate at a 1000 µm gap because the rigid gel could not be loaded reproducibly at the smaller gap. No pre-shear was applied. Cross-hatched (serrated) plates were used throughout to reduce wall slip, a known concern in parallel-plate measurements of viscoplastic gels [39]. Samples were allowed to relax for 5 min after loading and trimming, and a fresh aliquot was taken from the product for each test type, so that no sample was subjected to more than one measurement.

A single amplitude sweep (0.01–100% strain, 6.283 rad s^−1^) was recorded per formulation as an operational pre-test to select the strain for the frequency sweeps. Frequency sweeps were then run from 0.1 to 100 rad s^−1^ at 1.0% strain for K1, K2, M1, M2, L1, L2, G1 and G2, and at 0.5% strain for the κ-carrageenan formulations C1 and C2, whose linear region was markedly narrower; for C2 the applied strain of 0.5% lay about three times above its strict linear limit of 0.16%. *G′*, *G″*, tan *δ* and complex viscosity were recorded, and the frequency dependence of the storage modulus was expressed as the exponent *n′* of the power law

(1) *G′(ω) = G′_0_ω^n′^*

where *G′*_0_ (Pa) is the storage modulus extrapolated to *ω* = 1 rad s^−1^ and *n′* is the dimensionless frequency-dependence exponent. The exponent was obtained by linear regression of log *G′* on log *ω* over the full measured window, fitted separately to each replicate. Flow sweeps covered 0.1 to 100 s^−1^, recording shear stress, apparent viscosity and normal force. Flow curves were described by the Ostwald–de Waele (power-law) model

(2) $$\sigma =K\gamma^{\overset{\cdot }{n}}$$

and by the Herschel–Bulkley model

(3) $$\sigma =\tau_{0}K\gamma^{\overset{\cdot }{n}}$$

where *σ* (Pa) is the shear stress, $\overset{\cdot }{\gamma }$ (s^−1^) the shear rate, *τ*_0_ (Pa) the yield stress, *K* (Pa s*^n^*) the consistency index and n the flow behavior index; *K* and *n* are model-specific, and the model selected for each formulation is given in Table 3. Power-law parameters were obtained by linear regression of log *σ* on log $\overset{\cdot }{\gamma }$ and Herschel–Bulkley parameters by constrained non-linear least squares (*τ*_0_ ≥ 0, 0 < *n* < 1). For C2, which was progressively expelled from the gap at higher shear rates, the analyzed interval was truncated at the shear rate below which the normal force remained within 20% of its plateau value in all three replicates. Three independent pudding batches were measured per formulation.

### 4.8. Differential Scanning Calorimetry

Thermal properties were determined with a DSC 250 (TA Instruments, New Castle, DE, USA) fitted with an RCS 90 refrigerated cooling system. Samples weighing 11.4–14.4 mg were sealed in Tzero hermetic aluminum pans, with an empty hermetic pan used as the reference. Thermal analysis was performed on the three independent pudding batches produced for each formulation, with one pan prepared per batch; for C1, one batch was additionally measured in technical duplicate. Samples were equilibrated at 5 °C and held for 5 min, heated to 100 °C at 10 °C min^−1^ and held for 2 min, cooled to 5 °C at 10 °C min^−1^ and held for 2 min, then reheated to 100 °C at 10 °C min^−1^. Nitrogen purge flow was 50 mL min^−1^.

The peak temperature (T_p_) was first located on the smoothed first-heating curve, and the integration window was then centered on it (T_p_ ± 10 °C), with a linear baseline anchored to the 5 °C bands immediately preceding and following this window; the same procedure was applied to all formulations. The onset temperature (T_o_) was taken as the intersection of the inflection tangent with the extrapolated baseline. Noise was defined as the residual standard deviation of a linear fit over 25–45 °C, a region in which no transition is expected, and a transition was regarded as resolved only where the baseline-corrected signal exceeded three times this value. The sensitivity of the reported metrics to the width of the integration window was assessed by varying the half-width between 8 and 12 °C. Curves were excluded if the baseline heat flow changed by more than 5% between the first and second heating scans, indicating loss of water from the pan, or if the baseline drift within a scan exceeded twice that of the remaining curves of the same formulation. Apparent enthalpies are reported both per gram of sample and per gram of κ-carrageenan.

### 4.9. ATR-FTIR Spectroscopy

Fresh puddings were analyzed after the 12 h refrigerated set in ATR mode on a Shimadzu IRAffinity spectrometer (Shimadzu Corporation, Kyoto, Japan) fitted with a diamond ATR crystal. Spectra were collected from 4000 to 400 cm^−1^ at 4 cm^−1^ resolution by averaging 48 scans per sample. Three independent pudding batches were analyzed per formulation, and the three batch spectra were averaged to form the formulation-level mean. No penetration-depth (ATR) correction, baseline correction, or derivative transformation was applied to the reported spectra because all spectra were acquired on the same instrument under identical conditions; the wavelength dependence of the penetration depth is common to them and cancels in the between-formulation comparison. Spectra were normalized on the O–H stretching envelope before averaging. Differences between formulations were quantified over the 800–1300 cm^−1^ polysaccharide region as the mean absolute difference between vector-normalized group means. They were judged against the mean absolute dispersion between replicates of the same formulation. Second-derivative spectra (Savitzky–Golay, 13 points, third order) were computed as a supplementary check only. Smoothing was applied to Figure 5 for display purposes alone and not to any spectrum used in the numerical comparison.

### 4.10. Statistical Analysis

The independently produced pudding batch was the experimental unit, and all results are reported as the mean ± standard deviation from three independent productions unless stated otherwise. Moduli and viscosities were log_10_-transformed before analysis. Because group variances were markedly heterogeneous, differences among the ten formulations were assessed using a Welch one-way analysis of variance, followed by Games–Howell pairwise comparisons, with Kruskal–Wallis tests as omnibus sensitivity analyses; homogeneous groups are indicated by superscript letters in the tables. The eight gum-structured formulations were additionally analyzed as a balanced two-factor design (four gum types × two concentrations), including the interaction term, with partial η^2^ reported as the effect size. Planned contrasts comparing the two concentrations within each gum and the K1–K2 control contrast were evaluated using Welch t-tests with Holm adjustment for each outcome. Pearson correlation, principal component analysis of the standardized rheological descriptors, and hierarchical clustering (Ward’s method, Euclidean distance) were applied to the formulation means; correlation coefficients were tested for significance, while the ordination and clustering summarize the covariance structure of the data set. DSC and FTIR results were treated descriptively. Significance was set at α = 0.05. All analyses were performed in R version 4.6.1 (R Core Team, Vienna, Austria).

## Figures and Tables

**Table 1:** Replicate gravimetric determination of the moisture and dry-matter contents of Kırkağaç melon seeds after pre-drying at 40 °C for 24 h.

Replicate	Initial Sample (g)	Dry Sample (g)	Dry Matter (%)	Moisture (%)
1	4.1275	2.4170	58.56	41.44
2	4.0510	2.6423	65.23	34.77
3	4.0080	2.5307	63.14	36.86
Mean ± SD			62.31 ± 3.41	37.69 ± 3.41

**Table 2:** Viscoelastic parameters of the pudding formulations obtained from frequency sweeps at a reference angular frequency of 1 rad s^−1^, together with the frequency dependence of the storage modulus over 0.1–100 rad s^−1^ (25 °C; mean ± SD, *n* = 3 independent productions).

Sample	*G′* (Pa)	*G″* (Pa)	tan *δ*	η* (Pa·s)	*n′*	*R^2^*	*ω*_c_ (rad s^−1^)
K1	149.92 ± 40.51 ^bcd^	17.60 ± 3.80 ^de^	0.119 ± 0.011 ^e^	150.96 ± 40.66	0.097 ± 0.016	0.994	
K2	396.11 ± 85.14 ^ab^	53.45 ± 11.45 ^bc^	0.135 ± 0.003 ^e^	399.70 ± 85.90	0.157 ± 0.017	0.992	
M1	18.12 ± 1.07 ^fg^	5.38 ± 0.27 ^f^	0.297 ± 0.003 ^dg^	18.90 ± 1.10	0.195 ± 0.009	0.979	
M2	54.49 ± 2.70 ^de^	13.47 ± 0.61 ^e^	0.247 ± 0.001 ^f^	56.13 ± 2.76	0.163 ± 0.003	0.990	
L1	15.69 ± 8.84 ^cdefgh^	6.96 ± 3.73 ^bcdef^	0.452 ± 0.035 ^d^	17.16 ± 9.59	0.308 ± 0.004	0.996	
L2	15.76 ± 1.64 ^g^	26.44 ± 1.37 ^cd^	1.684 ± 0.087 ^a^	30.79 ± 2.02	0.752 ± 0.025	0.967	5.14
C1	359.79 ± 27.37 ^abc^	81.05 ± 15.79 ^ab^	0.224 ± 0.031 ^efg^	368.92 ± 29.69	0.126 ± 0.013	0.833	
C2	475.40 ± 116.53 ^a^	236.01 ± 78.17 ^a^	0.495 ± 0.096 ^cdefg^	531.73 ± 134.70	0.213 ± 0.019	0.780	0.18
G1	3.68 ± 0.39 ^h^	4.68 ± 0.23 ^f^	1.276 ± 0.068 ^b^	5.96 ± 0.42	0.632 ± 0.022	0.959	2.12
G2	33.78 ± 5.18 ^ef^	27.48 ± 3.05 ^cd^	0.817 ± 0.032 ^c^	43.55 ± 5.95	0.446 ± 0.014	0.940	0.53

K1 and K2 denote the starch-structured melon seed beverage and cow-milk controls, respectively; M, L, C and G denote xanthan gum, locust bean gum, κ-carrageenan and guar gum, respectively; suffixes 1 and 2 denote concentrations of 0.5 and 1.0% (w/w), respectively. Within each column, means not sharing a superscript letter differed significantly (Games–Howell, *p* < 0.05). *n′*, exponent of the power-law fit of *G′* against angular frequency over 0.1–100 rad s^−1^, fitted separately to each replicate and reported as mean ± SD; *R*^2^, mean coefficient of determination of the three individual log–log fits; *ω*_c_, mean angular frequency of the *G′–G″* crossover, determined separately in each replicate and observed in all three where reported, and absent throughout the window elsewhere. A lower exponent denotes a storage modulus that depends less on frequency and therefore a network that persists over a wider range of timescales; the exponent is used as a relative ranking rather than a categorical criterion. Oscillatory values for C2 should be interpreted with caution, because the applied strain exceeded its strict linear viscoelastic limit; C2 is nevertheless retained in the factorial and multivariate analyses.

**Table 3:** Steady-shear flow parameters of the pudding formulations at 25 °C (mean ± SD, *n* = 3 independent productions).

Sample	Model	η at 1 s^−1^ (Pa·s)	*τ*_0_ (Pa)	K (Pa·s*^n^*)	*n*
K1	PL	36.37 ± 3.57 ^c^		37.16 ± 3.65	0.297 ± 0.031
K2	PL	159.10 ± 36.86 ^a^		150.09 ± 33.33	0.262 ± 0.008
M1	PL	11.60 ± 0.11 ^efg^		10.84 ± 0.13	0.133 ± 0.004
M2	PL	20.11 ± 0.97 ^bd^		20.01 ± 0.73	0.194 ± 0.001
L1	PL	16.76 ± 1.14 ^def^		15.59 ± 1.03	0.357 ± 0.021
L2	PL	28.32 ± 3.04 ^bc^		24.94 ± 2.59	0.550 ± 0.009
C1	n.d.	38.68 ± 6.06 ^bc^	n.d.	n.d.	n.d.
C2	HB	11.69 ± 1.31 ^f^	6.76 ± 1.73	4.76 ± 0.77	0.529 ± 0.128
G1	PL	6.31 ± 0.77 ^g^		5.36 ± 0.68	0.471 ± 0.014
G2	PL	37.04 ± 7.70 ^bcde^		31.28 ± 5.72	0.327 ± 0.034

PL, Ostwald–de Waele (power-law) model; HB, Herschel–Bulkley model; *τ*_0_, yield stress; K, consistency coefficient; *n*, flow behavior index. Different superscript letters in the viscosity column denote significant differences (Games–Howell, *p* < 0.05). C2 was evaluated over the common interval within which the normal force remained within 20% of its plateau in all replicates (0.1–1.59 s^−1^). n.d., not determined: for C1, the shear stress was almost independent of shear rate, so that neither model provided an identifiable parameterization (see Section 2.5). Because *K* and *n* carry different meanings in the two models, they are compared only within their respective model classes.

**Table 4:** Two-factor analysis of variance for the eight gum-structured formulations (four gum types × two concentrations, *n* = 3 per cell). The starch controls K1 and K2 were excluded.

Outcome	Effect	F	df	*p*	Partial η^2^
log_10_ *G′*	Gum type	271.30	3, 16	<0.001	0.981
	Concentration	85.72	1, 16	<0.001	0.843
	Gum × concentration	23.31	3, 16	<0.001	0.814
log_10_ *G″*	Gum type	166.37	3, 16	<0.001	0.969
	Concentration	170.04	1, 16	<0.001	0.914
	Gum × concentration	3.79	3, 16	0.031	0.416
tan *δ*	Gum type	360.05	3, 16	<0.001	0.985
	Concentration	120.42	1, 16	<0.001	0.883
	Gum × concentration	253.11	3, 16	<0.001	0.979
log_10_ η at 1 s^−1^	Gum type	16.06	3, 16	<0.001	0.751
	Concentration	68.40	1, 16	<0.001	0.810
	Gum × concentration	150.14	3, 16	<0.001	0.966

**Table 5:** Baseline-corrected DSC transition metrics of the κ-carrageenan formulations. No transition was resolved in any other formulation (mean ± SD across independent productions).

Formulation	Evaluable/Total Independent Productions	T_o_ (°C)	T_p_ (°C)	Δ*H* (J g^−1^ sample)	Δ*H* (J g^−1^ κ-carrageenan)
C1 (κ-carrageenan 0.5%)	3/3	65.1 ± 2.6	68.2 ± 3.3	0.42 ± 0.19	84 ± 38
C2 (κ-carrageenan 1.0%)	1/3	75.4	77.2	0.29	29
All other formulations	3/3	not resolved	not resolved	<0.05	

T_o_, onset temperature; T_p_, peak temperature; Δ*H*, apparent enthalpy of the resolved thermal transition. Entries in the evaluable/total column refer to independent pudding productions. Three independent productions were analyzed for each formulation. For C2, only one of the three thermograms met the predefined quality criteria and was therefore evaluable. The other two C2 pans showed evidence of water loss during the first heating scan, with a broad endotherm centered between 52 and 60 °C and a 10 and 21% reduction in baseline heat flow between the first and second heating scans. The pans showed no visible damage, and the loss was attributed to partial venting of the hermetic seal under internal vapor pressure; these two curves were excluded. One C1 curve was excluded because its baseline drift exceeded twice that of the remaining curves. The excluded curve was one of a technical duplicate pair; therefore, the reported C1 values represent one evaluable curve from each of the three independent productions. The technical duplicate was not counted as an additional independent production. The four retained κ-carrageenan curves showed no change in baseline heat flow between heating scans. Signal-to-noise ratios were 23 for C1 and 30 for C2, compared with 1.5 for the control window. Varying the integration half-width between 8 and 12 °C changed Δ*H* by 18% for C1 and 62% for C2.

## Data Availability

The original contributions presented in this study are included in the article. Further inquiries can be directed to the corresponding author.

## Abbreviations

The following abbreviations are used in this manuscript:

Abbreviation	Definition
ATR-FTIR	Attenuated total reflectance Fourier-transform infrared spectroscopy
DSC	Differential scanning calorimetry
*G′*	Storage modulus
*G″*	Loss modulus
tan *δ*	Loss tangent (*G″*/*G′*)
η*	Complex viscosity
*n′*	Power-law exponent of the storage modulus against angular frequency
*ω*_c_	Angular frequency of the *G′*-*G″* crossover
*τ*_0_	Yield stress
K	Consistency coefficient
n	Flow behavior index
PL	Ostwald–de Waele (power-law) model
HB	Herschel–Bulkley model
S/N	Signal-to-noise ratio
LBG	Locust bean gum
LVR	Linear viscoelastic region
PCA	Principal component analysis
PDO	Protected designation of origin
w/w	Weight by weight
